# Pre-hospital Triage of Acute Ischemic Stroke Patients—Importance of Considering More Than Two Transport Options

**DOI:** 10.3389/fneur.2019.00437

**Published:** 2019-04-26

**Authors:** Ludwig Schlemm, Eckhard Schlemm, Christian H. Nolte, Matthias Endres

**Affiliations:** ^1^Department of Neurology, Charité – Universitätsmedizin Berlin, Corporate Member of Freie Universität Berlin and Humboldt-Universität zu Berlin, and Berlin Institute of Health (BIH), Berlin, Germany; ^2^Center for Stroke Research Berlin (CSB), Charité – Universitätsmedizin, Berlin, Germany; ^3^Berlin Institute of Health (BIH), Berlin, Germany; ^4^Medizinische Fakultät, Universität Hamburg, Hamburg, Germany; ^5^Klinik und Poliklinik für Neurologie, Kopf- und Neurozentrum, Universitätsklinikum Hamburg-Eppendorf, Hamburg, Germany; ^6^DZHK (German Center for Cardiovascular Research), Partner Site, Berlin, Germany; ^7^DZNE (German Center for Neurodegenerative Diseases), Partner Site, Berlin, Germany

**Keywords:** pre-hospital triage, decision analysis, ischemic stroke, thrombolysis, mechanical thrombectomy, health services research, geospatial modeling, mathematical modeling (medical)

## Abstract

**Background:** Patients with acute ischemic stroke (AIS) and large vessel occlusion benefit from rapid access to mechanical thrombectomy in addition to intravenous thrombolysis. Prehospital triage algorithms to determine the optimal transport destination for AIS patients with unknown vessel status have so far only considered two alternatives: the nearest comprehensive (CSC) and the nearest primary stroke center (PSC).

**Objective:** This study explores the importance of considering a larger number of PSCs during pre-hospital triage of AIS patients.

**Methods:** Analysis was performed in random two-dimensional abstract geographic stroke care infrastructure environments and two models based on real-world geographic scenarios. Transport times to CSCs and PSCs were calculated to define sub-regions with specific triage properties. Possible transport destinations included the nearest CSC, the nearest PSC, and any of the remaining PSCs that are not closest to the scene, but transport to which would imply a shorter total time-to-CSC-via-PSC.

**Results:** In abstract geographic environments, the median relative size of the sub-region where a triage decision is required ranged from 34 to 92%. The median relative size of the sub-region where more than two triage options need to be considered ranged from 0 to 56%. The achievable reduction in time-to-thrombectomy (“benefit”) exceeded the increase in time-to-thrombolysis (“harm”) by a factor of 2 in 30.5–37.0% of the sub-region where more than two triage options need to be considered. Results were confirmed in geographic environments based on real-world urban and rural stroke care infrastructures.

**Conclusion:** Pre-hospital triage algorithms for AIS patients that only take into account the nearest CSC and the nearest PSC as transport destinations may be unable to identify the optimal transport destination for a significant proportion of patients.

## Introduction

### Background

International guidelines recommend early administration of intravenous thrombolysis for eligible patients with acute ischemic stroke (AIS); in addition, patients with proximal large vessel occlusion (LVO) should receive mechanical thrombectomy (MT) as quickly as possible (1). As the clinical benefit of both thrombolysis (2–4) and MT (5–7) diminishes over time, research efforts in recent years have focused on improving clinical outcome by reducing pre-hospital (8–10) and intra-hospital delays (11, 12). With regard to pre-hospital delays, directly transporting AIS patients to an MT-capable comprehensive stroke center (CSC) instead of a nearer non-MT-capable primary stroke center (PSC) has been suggested as one strategy to reduce time to MT for patients with LVO (13). Given that information about the vessel status of patients is typically not available to emergency medical personnel in the field, patients that are likely to benefit from direct transportation to a CSC need to be selected based on clinical and demographic variables. Several clinical pre-hospital stroke severity scales with similar accuracies to estimate the likelihood of LVO exist (14); however, the optimal instruments as well as the most appropriate cutoff values to inform pre-hospital triage decisions and to select patients for direct transportation to a CSC are not currently known (1). Previous studies that explored the impact of triage algorithms to determine the most adequate transport destination for AIS patients only allowed for a decision between two alternatives, namely transport to the nearest CSC, bypassing all PSCs; and transport to the nearest PSC (15–17). However, clinical experience as well as fundamental geographic observations suggest that oftentimes a PSC that is not nearest to the scene, but from which a patient could be transferred quickly to a CSC if necessary, might be a better primary transport destination option than the nearest PSC.

### Objective

In the current study, we aimed to evaluate the importance of considering all possible transport destinations during pre-hospital triage of AIS patients beyond the established “directly to CSC vs. nearest PSC” paradigm.

## Methods

### Model

The importance of extending the array of possible transport destinations was explored in abstract two-dimensional geographic stroke care environments and subsequently in two different real-world geographies. For the first part of the analysis, we used computational simulations to generate independent random realizations of abstract geographic environments characterized by different numbers of PSCs (2–10) and CSCs (1–4) that could be reached within established treatment time windows. The range of the number of stroke centers was chosen to reflect different stroke care infrastructure environments while considering computational efficiency. PSCs were assumed to be comparable in terms of door-to-needle and door-in-door-out time. Locations of stroke centers were determined by a homogenous spatial Poisson point process in the disc. The base unit of the model was taken as driving time under current weather and traffic condition which were assumed homogenous within the incident region. To explore the impact of different spatial stroke center densities, two models with different radius of the disc (30 and 120 min) were analyzed separately. The travel time from any point within the incident region to any CSC or PSC was calculated as the Euclidean distance between the two locations.

For the second part of the analysis, real-world geographic scenarios with stroke center distributions and traffic network infrastructures based on those of Berlin, Germany (metropolitan region) and those of Brandenburg, Germany (mixed rural region) were used. Since an unusually high proportion of stroke centers in the city of Berlin offer MT-services (up to 11 of 14, depending on the time of day), we limited the availability of MT-services in the analysis of the urban scenario to three university hospitals (see Supplementary Data for a detailed description of the two regions). Distances between points were determined with the haversine formula and transformed into driving times using the Google Maps Distance Matrix API.

### Definition of Sub-regions and Benefit/Harm Ratio

For all scenarios, sub-regions were defined according to specific patterns of travel times from the points within the sub-region to the CSCs and PSCs. All locations at which the closest stroke center was a CSC and thus only one reasonable transport destination exists were defined as unconditional catchment regions of a CSC. We defined the triage region as all points where the nearest stroke center was not a CSC. This region was further sub-divided according to the number of transport destinations that need to be considered for an exhaustive triage decision. Potential transport destinations included the nearest CSC, the nearest PSC, and, in addition, any of the remaining PSCs that are not closest to the scene, but transport to which would imply a shorter total time-to-CSC-via-PSC as compared to transport to any other PSC that is closer to the scene. The higher order triage region was defined as all points where more than two possible transport destination existed (see Supplementary Data for formal mathematical definitions).

**Figure 1:**
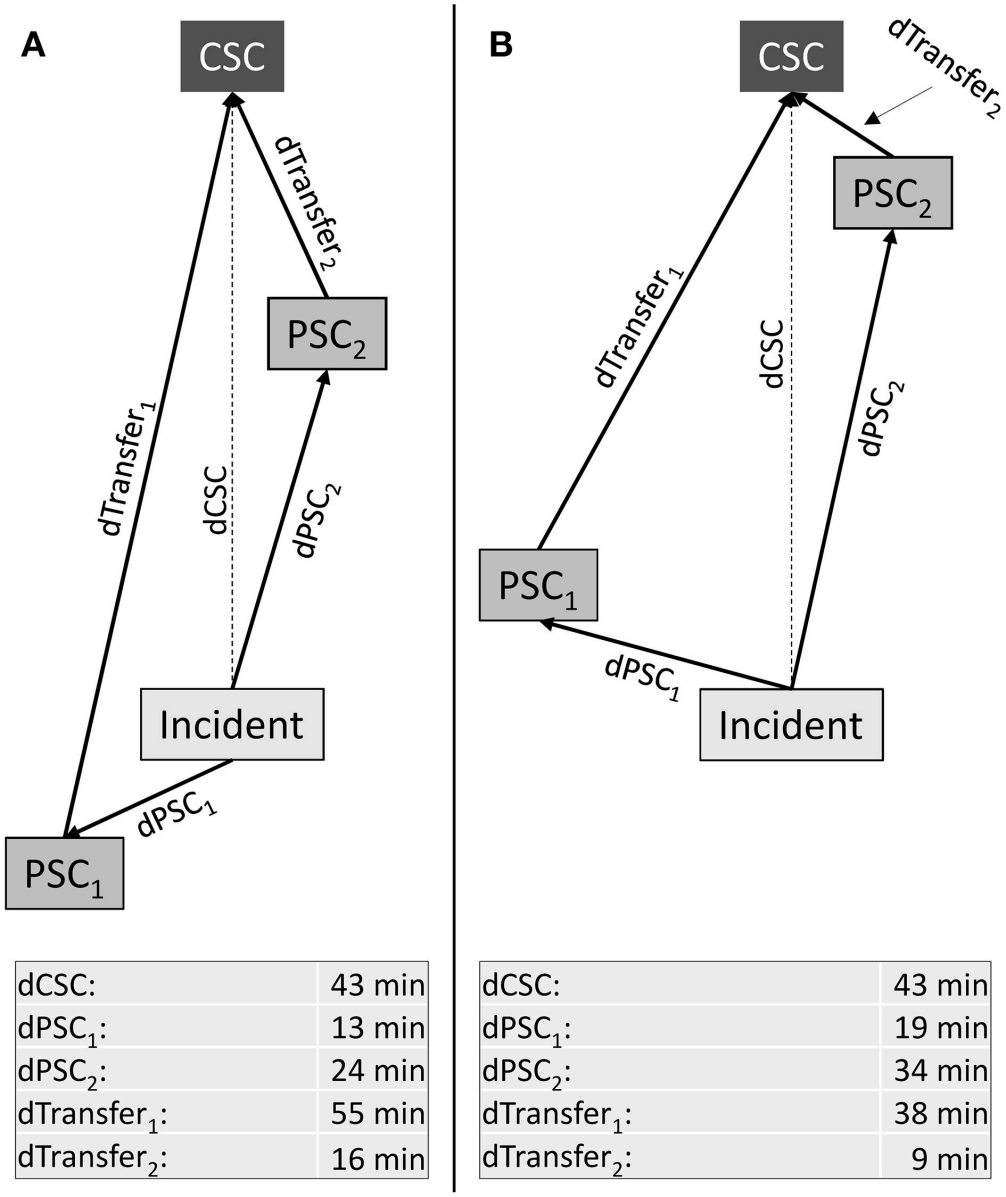
Higher order triage with three potential transport destinations. Abstract geographic scenarios with one comprehensive (CSC) and two primary stroke centers (PSC). Lengths of arrows displayed in the table at the bottom represent driving time. Transport time from the location of the stroke incident to the CSC is larger than to any one of the two PSCs. Additionally, primary transport to the nearest PSC (PSC_1_) is associated with longer transport time to the CSC for patients requiring secondary transfer as compared to primary transport to PSC_2_. The benefit/harm ratios (BHR) associated with considering PSCs that are not nearest to the scene (in this example: PSC_2_) can be calculated (1) in relation to the patient being transported to the nearest PSC; and (2) in relation to the patient being transported directly to the nearest CSC. For (1), it is the ratio of the reduction in time-to-MT and the increase in time-to-thrombolysis; for (2), the ratio of the reduction in time-to-thrombolysis and the increase in time-to-MT: $BHR_{PSC}=\frac{\left(dPSC_{1}+dTransfer_{1}\right)-\left(dPSC_{2}+dTransfer_{2}\right)\;}{dPSC_{2}-\;dPSC_{1}}.$
$BHR_{CSC}=\frac{dCSC-\;dPSC_{2}}{\left(dPSC_{2}+{DIDO+dTransfer}_{2}\right)-dCSC}.$ **(A):** Nearest PSC (PSC_1_) and CSC are in opposite directions from the scene while another PSC that is not nearest to the scene (PSC_2_) lies in direction toward the CSC: $BHR_{PSC}=\frac{\left(13+55\right)-\left(24+16\right)\;}{24-\;13}=2.54,$
$BHR_{CSC}=\frac{43-24}{\left(24+60+16\right)-\;43}=\;0.33.$ **(B):** Both PSCs lie in direction toward the CSC: $BHR_{PSC}=\frac{\left(19+38\right)-\left(34+9\right)\;}{34-\;19}=0.93$,
$$BHR_{CSC}=\frac{43-34}{\left(34+60+9\right)-\;43}=\;0.15$$.

To quantify the potential clinical usefulness of considering additional PSCs as primary transport destinations, for each point within the higher order triage region, we calculated two types of ratios: (1) the ratio of the reduction in time-to-MT (“benefit”) and the increase in time-to-thrombolysis (“harm”) associated with primary transport to each additionally considered PSC as compared to primary transport to the nearest PSC. And (2) the ratio of the reduction in time-to-thrombolysis (“benefit”) and the increase in time-to-MT (“harm”) associated with primary transport to each additionally considered PSC as compared to primary transport to the nearest CSC. The greatest of the ratios between the so determined benefits and harms for each point within the higher order triage region was defined as surrogate parameter of the potential clinical usefulness of considering additional PSCs (Figure 1). Calculation of the benefit/harm ratio in relation to the nearest CSC required specification of a door in-door out time, which was assumed to be 60 min and varied in sensitivity analyses.

### Analysis

For the statistical analysis of abstract geographic scenarios, 50 random geographic stroke care environments were simulated for each combination of PSCs and CSCs. Results are presented as full distributions or as summary statistics with boxplots showing the median and interquartile ranges (IQR). All simulations and analyses were performed in MATLAB (18).

No ethical approval and no informed consent was required for this study. The MATLAB code of our project is publicly available on GitHub (https://github.com/lschlemm/Higher-Order-Effects-of-Prehospital-Triage).

## Results

### Random Scenarios

**Figure 2:**
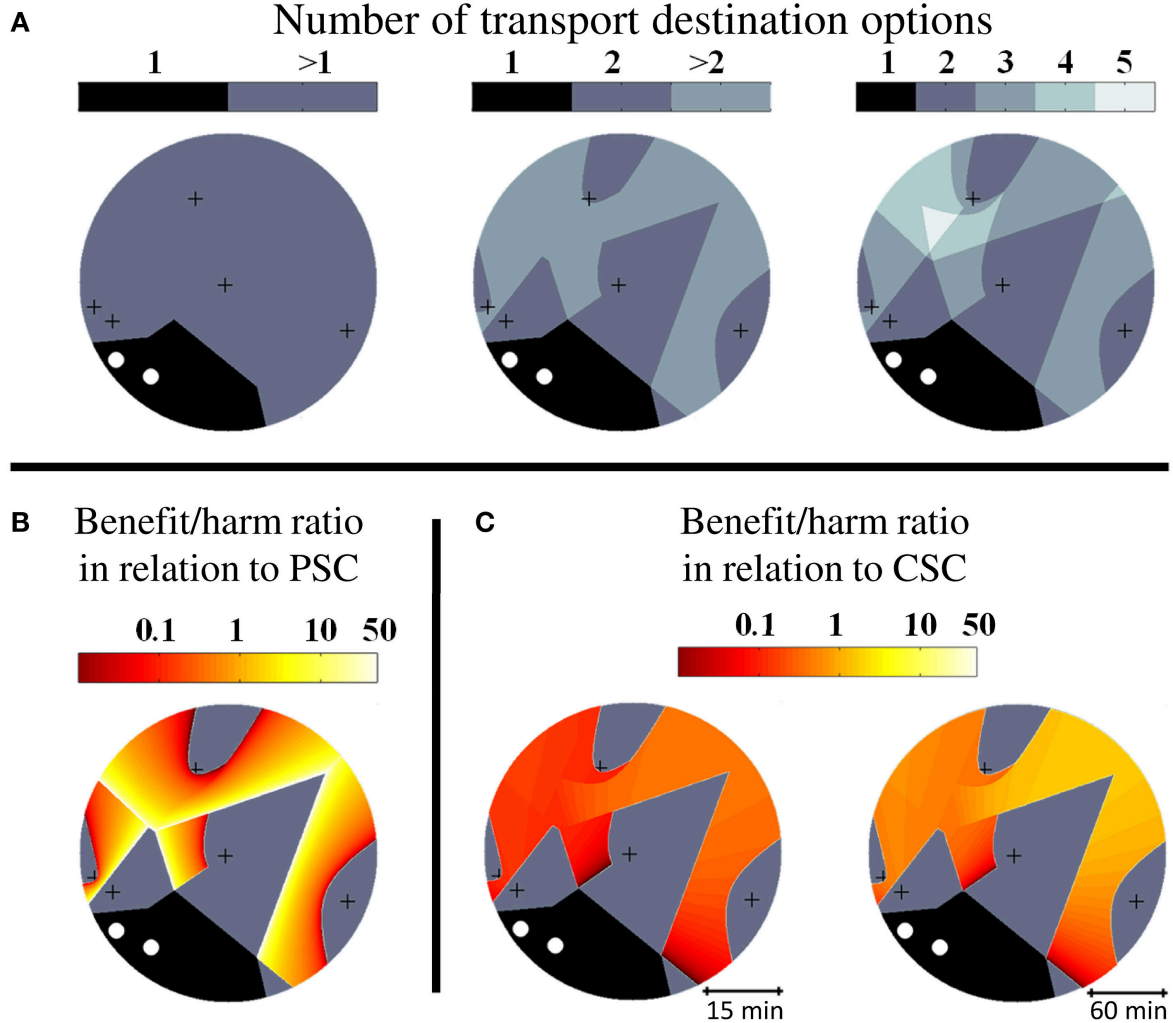
Higher order triage in abstract geographic environments. Visualization of triage sub-regions and benefit/harm ratios in exemplary abstract geographic scenarios with two comprehensive stroke centers (CSC, white circles) and five primary stroke centers (PSC, black crosses). **(A):** Diagram on the left shows the extent of the unconditional catchment region of the CSCs (1) and the extent of the region where more than one possible transport destination exist and hence a triage decision is required (>1). The diagram in the middle highlights the sub-region where more than two triage options need to be considered (>2). The diagram on the right shows the subdivision of the higher order triage region according to the number of transport destination options (3–5). **(B):** Spatial distribution of the benefit/harm ratio in relation to the nearest PSC. **(C):** Spatial distribution of the benefit/harm ratio in relation to the nearest CSC.

In a first step, we created random geographic stroke care environments with different numbers of CSCs and PSCs and determined the regions with characteristic properties relating to pre-hospital triage decisions. An exemplary geographic setting with two CSCs and five PSCs together with graphical illustrations of the size and distribution of the sub-regions and the benefit/harm ratios is shown in Figures 2A–C. In geographic settings with two CSCs and five PSCs, the median benefit/harm ratio calculated in relation to the nearest PSC was 1.02 (IQR 0.81–1.11). Since transportation of a patient to a PSC instead of a CSC involves an additional secondary transfer, the median benefit/harm ratio calculated in relation to the nearest CSC was lower than the benefit/harm ratio calculated in relation to the nearest PSC and depended on the average driving time between stroke centers (incident region with a radius of 30 min: median 0.13, IQR 0.09–0.18; incident region with a diameter of 120 min: median 0.56, IQR 0.35–0.77).

**Figure 3:**
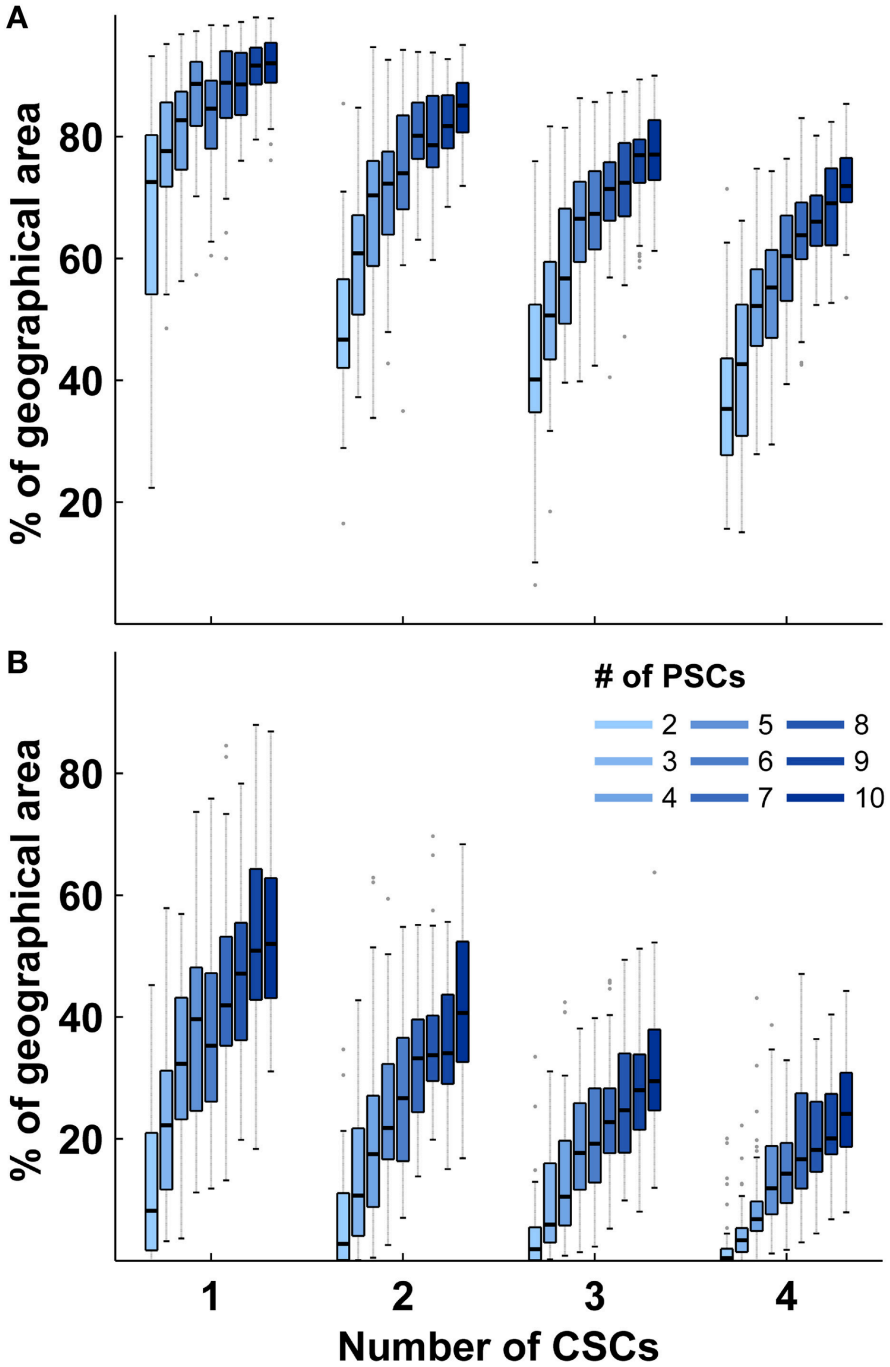
Relative sizes of triage sub-regions in abstract geographic environments. **(A):** Distribution of the relative size of region where a triage decision is required in relation to the total size of the geographic region according to the number of comprehensive and primary stroke centers. **(B):** Distribution of the relative size of region where more than two transport destinations need to be considered in relation to the total size of the geographic region according to the number of comprehensive and primary stroke centers. Shown are the median and interquartile ranges (IQR); length of whiskers correspond to 1.5-times the IQR. PSCs indicates primary stroke center; CSC, comprehensive stroke center.

**Figure 4:**
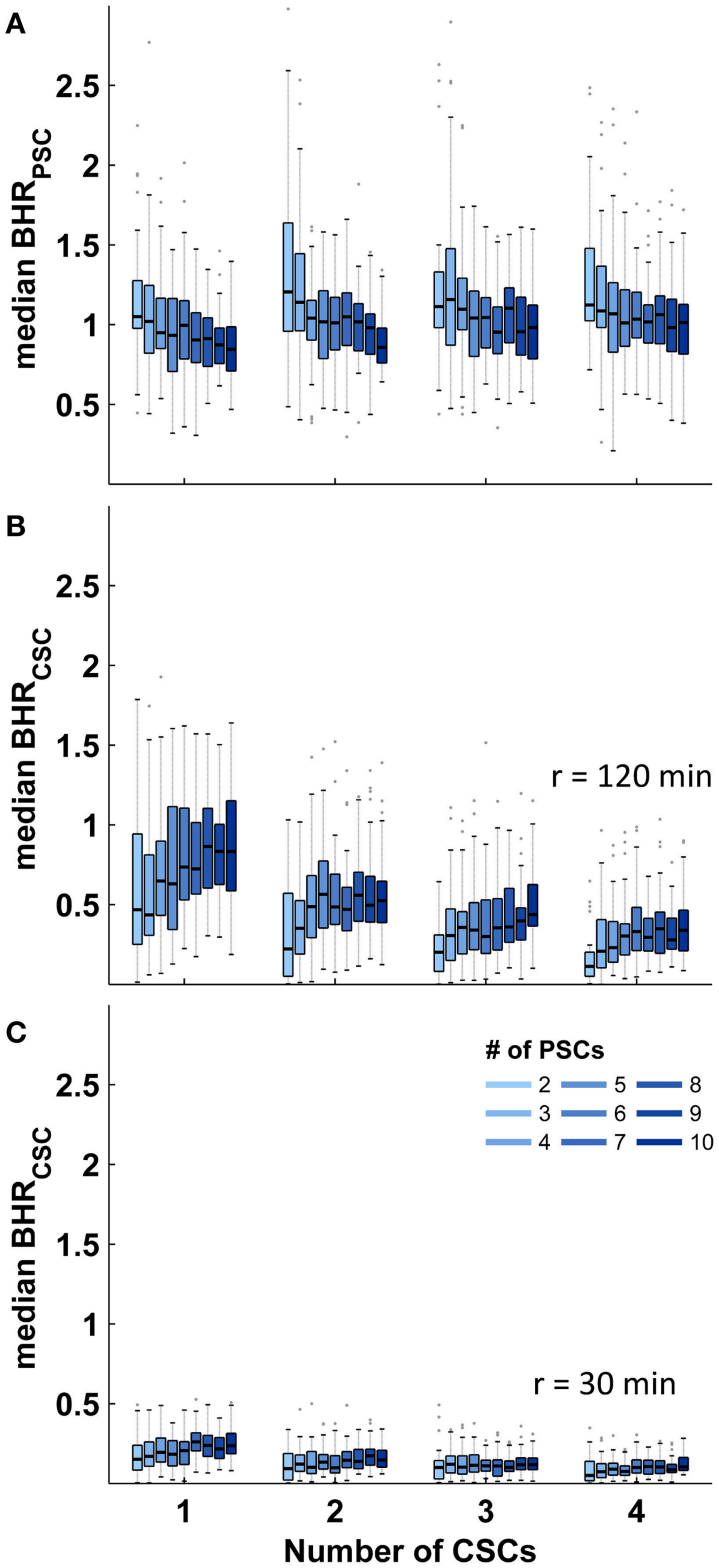
Benefit/harm ratios in abstract geographic environments. Distribution of the spatial median of the benefit/harm ratios (BHR) in regions where more than two transport destinations need to be considered according to the number of primary (PSCs) and comprehensive stroke centers (CSCs). **(A):** Benefit/harm ratio in relation to the nearest PSC. **(B,C):** Benefit/harm ratio in relation to the nearest CSC. Shown are the median and interquartile ranges (IQR); length of whiskers correspond to 1.5-times the IQR.

Identical calculations to those presented for stroke care infrastructure environments with two CSCs and five PSCs were performed for all combinations of different numbers of CSC: 1–4 and numbers of PSC: 2–10. The relative size of the triage region where a triage decision is required and the relative size of the higher order triage region where more than two triage options need to be considered depended on both the total number of CSCs and the total number of PSCs with medians ranging from 34 to 92% and 0 to 56%, respectively. The relative sizes of both regions showed a negative correlation with the total number of CSCs and a positive non-linear association with the total number of PSCs (Figure 3). Across all geographic scenarios, the median of the benefit/harm ratio calculated in relation to the nearest PSC was 1.01 (IQR 0.84–1.19) with slightly higher values in geographic scenarios with fewer PSCs (Figure 4A). The reduction in time-to-MT exceeded the increase in time-to-thrombolysis by a factor of at least 2 in 30.5–37.0% of the area of the higher order triage region As expected, the benefit/harm ratio calculated in relation to the nearest CSC was lower (incident region with a radius of 30 min: median 0.13, IQR 0.08–0.20; incident region with a radius of 120 min: median 0.41, IQR 0.25–0.67) with slightly higher values in geographic scenarios with more PSCs (Figures 4B,C). Similar results were obtained assuming a door in-door-out time of 30 and 90 min.

### Real World Scenarios

**Figure 5:**
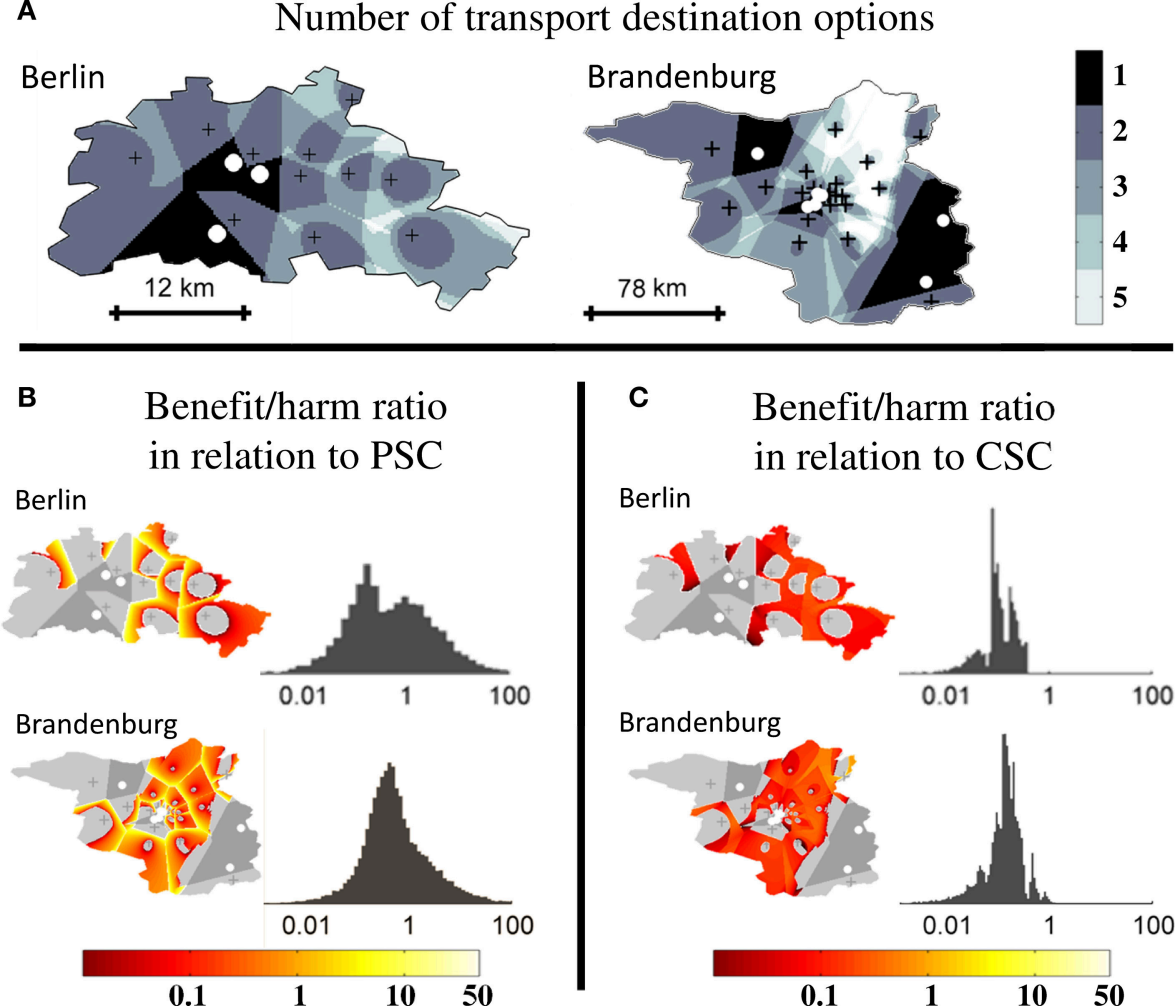
Higher order triage in real-world geographic scenarios. Extent of triage sub-regions and benefit/harm ratios in a real-world urban (Berlin) and rural (Brandenburg) geography. Comprehensive stroke centers (CSC) are marked with white circles and primary stroke centers (PSC) with black crosses. **(A):** Regional subdivision according to the number of transport destination options. **(B):** Spatial distribution and frequency distribution of the benefit/harm ratio in relation to the nearest PSC. **(C):** Spatial distribution and frequency distribution of the benefit/harm ratio in relation to the nearest CSC.

To confirm validity of the results obtained in abstract geographic environments, analyses were also performed in two scenarios based on the geographic stroke care infrastructure environments of Berlin (urban) and Brandenburg (rural). In the urban environment, the relative size of the triage region where a triage decision is required and the relative size of the higher order triage region where more than two triage options need to be considered were 84% and 35%, respectively. In the rural environment, relative sizes were 81% and 52%, respectively (Figure 5A). The order of magnitude and distribution of the benefit/harm ratios calculated in both real-world environments were comparable to those obtained in abstract geographic scenarios: the median benefit/harm ratios in relation to the nearest PSC in the urban and rural environment were 0.39 (IQR 0.10–1.92) and 0.57 (IQR 0.29–1.22), respectively. The reduction in time-to-MT exceeded the increase in time-to-thrombolysis by a factor of at least 2 in 21% of the area of the higher order triage region in the urban environment and in 12.9% in the rural environment. The benefit/harm ratios in relation to the nearest CSC were overall small, and lower in the urban environment (median 0.10. IQR 0.08–0.18) than in the rural environment (median 0.14. IQR 0.09–0.20), reflecting a greater influence of the door in-door out time in relation to transport times (Figures 5B,C).

## Discussion

### Main Findings

We analyzed the importance of considering all possible transport destinations during pre-hospital triage of AIS patients in abstract and real-world geographic stroke care environments. Our results suggest that more than two possible primary transport destinations exist for a significant proportion of geographic locations. At approximately one third of those, the reduction in time-to-MT associated with direct transportation of a patient to a PSC that is not nearest to the scene is at least twice as large as the increase in time-to-thrombolysis. As expected, the relative size of the sub-region where more than two triage options need to be considered depends on the number of PSCs and CSCs and is largest for environments with few CSCs and many PSCs.

### Previous Studies

To the best of our knowledge, no previous study has explored the impact of extending the array of stroke centers considered as primary transport destinations in pre-hospital triage algorithms beyond the established “directly to CSC vs. nearest PSC” paradigm. Increasingly, pre-hospital clinical stroke severity scales are being investigated as tools to identify patients with LVO that might benefit from direct transportation to a CSC (14); however, there is currently not enough evidence to recommend use of one specific scale in clinical practice (1). The results of our analysis have direct implications for the concept of pre-hospital triage of patients with suspected AIS: When the number of possible transport destinations exceeds two, decision support algorithms with a binary response, such as a fixed cutoff value of pre-hospital clinical stroke severity scales, are unsuitable for informing pre-hospital triage decisions. Instead, it is necessary to estimate the clinical outcome of individual patients given a set of clinical predictor variables, i.e., age, sex, comorbidities, and stroke severity (the latter operationalized by one of the available pre-hospital scales); the post-test probability of the presence of MT-treatable LVO; and the estimated delays to the initiation of thrombolysis and—if indicated—MT associated with each transport destination. It has previously been shown that estimation of clinical outcome parameters with conditional probabilistic models can be used to guide decisions regarding the optimal transport destination for AIS patients (16, 19). In addition, a proof-of-concept study involving abstract geographic scenarios with one single CSC and PSC demonstrated that use of variable clinical stroke severity scale-derived cut-offs values that take into account estimated transport times may lead to better clinical outcomes than fixed cutoff values (20).

### Clinical Implications

Not all patients suffering an ischemic stroke at a geographic location where more than two transport destinations exist would equally benefit from an exhaustive triage strategy: Patients with a low probability of LVO would only be expected to benefit at locations where the benefit/harm ratio in relation to the nearest PSC is high, while patients with more severe stroke symptoms and a higher probability of LVO would be expected to also derive benefit at locations with a lower benefit/harm ratio. Using the numerical values for the reduction in disability-adjusted life days per minute faster treatment published by Meretoja et al. (3, 7), the cut-off values for the benefit/harm ratio, above which a patient is expected to benefit from the consideration of higher order triage options, can be calculated as $\frac{1-pLVO}{pLVO}\times \frac{Effect_{Thrombolysis}}{Effect_{Thrombectomy}}$. For an estimated probability of LVO of 10, 30, and 50%, the cutoff values are 3.9, 1, and 0.4, respectively. Importantly, these cutoff values only apply if the patient would not be transferred directly to the nearest CSC in the first place.

In clinical practice, pre-hospital triage algorithms to determine the best transport destination for patients with suspected AIS are only needed if patients are eligible for both thrombolysis and MT. In addition, since no data are currently available regarding the impact of different triage destinations on the outcome of patients with stroke mimics and hemorrhagic strokes, decisions for patients with *suspected* AIS have to be made based on the expected outcome of patients with *definite* AIS. Accordingly, in our analysis, we focused on patients with definite AIS who, after careful assessment of indications and contraindications, would fulfill the eligibility criteria for both thrombolysis and MT. In future studies modeling the impact of pre-hospital triage strategies, if would be desirable to include data on the clinical outcome of patients with other diagnoses than AIS, such as hemorrhagic stroke, as a function of pre-hospital delay and type of hospital (PSC or CSC). Currently, however, such data are not available.

Depending on specific geographic circumstances and the relative locations of stroke centers to each other, implementation of pre-hospital triage algorithms can lead to a shift of AIS patients from PSCs to CSCs. In addition, triage algorithms that consider all potential transport destination options can also lead to increased number of AIS patients at certain PSCs. How these changes in the distribution of patient volumes affect quality of care in the short, medium, and long term (e.g., decrease due to reduction of experience at PSCs, or due to insufficient resources to cope with additional workload) was not addressed in the current study.

### Limitations

We are aware of the following limitations regarding the findings of our study. First, we assumed similar door-to-needle and door-in-door-out times across all PSCs. When using pre-hospital triage algorithms in clinical practice, best estimates for PSC-specific treatment time metrics derived from recent historical data collected at each PSC should be used. In addition, in the base-case scenario, a door in-door-out time of 60 min was used, which is shorter than what is currently observed in high-volume centers (21, 22). We decided to assume a shorter median door in-door out time to be able to investigate the effect of considering all potential transport destination options during pre-hospital triage in a health care system in which other modifiable factors contributing to treatment delay have been addressed. When a door in-door-out time of 30 and 90 min was assumed in univariate sensitivity analyses, results were similar to the base case scenario. In general, longer door in-door out times would leave the relative sizes of the triage sub-region as well as the benefit/harm ratio calculated in relation to the nearest PSC unaffected, and decrease the benefit/harm ratio calculated in relation to the nearest CSC. Second, part of the results were based on abstract geographical models in the two-dimensional plane; in such models involving a total number of points less than or equal to three, the distance between two points can be modeled to directly reflect the transport times between these two points. Since the determination of sub-regions and the calculation of potential clinical usefulness in our model involved comparison of distances between more than three points, our model requires the assumption of proportionality between geographic distances and transport times, i.e., the assumption of one single mode of transport and an overall homogenous quality of the transport infrastructure within the modeled area (e.g., roads, weather conditions). Our findings cannot be used to determine the optimal transport destination for a given patient; instead the results of our study indicate that consideration of more than two transport destinations may often be needed for an exhaustive pre-hospital triage decision. Due to the theoretical nature of this study, confirmation of the accuracy of our results in clinical settings is required.

### Conclusion

In conclusion, our results suggest that for a non-negligible number of AIS patients, more than two possible transport destinations exist and should be considered in pre-hospital triage algorithms. Depending on geographic circumstances and stroke severity, patients may have better clinical outcome when transported primarily to a PSC that is not closest to the location of the incident. Future studies should aim to develop and validate pre-hospital triage algorithms that predict clinical outcome based on patient-specific clinical data and that include stroke centers beyond the nearest CSC and the nearest PSC when determining the optimal transport destination for AIS patients.

## Author Contributions

LS and ES jointly conceived the study. LS reviewed the literature, developed the model, performed the simulations, analyzed and interpreted the data, and wrote the first draft of the manuscript. ES performed preliminary simulations and confirmed integrity of the final model. All authors reviewed and edited the manuscript for intellectual content and approved the final version of the manuscript.

### Conflict of Interest Statement

The authors declare that the research was conducted in the absence of any commercial or financial relationships that could be construed as a potential conflict of interest.
