# Supplementary material for: Pre-hospital Triage of Acute Ischemic Stroke Patients—Importance of Considering More Than Two Transport Options
Source: Front Neurol. 2019 Apr 26;10:437. doi: 10.3389/fneur.2019.00437 (PMC6503645; doi:10.3389/fneur.2019.00437)
Supplement: Supplementary file 1 [file Data_Sheet_1.docx]

ONLINE SUPPLEMENT

Prehospital Triage of Acute Ischemic Stroke Patients – Importance of Considering More than Two Transport Options

Ludwig Schlemm, Eckhard Schlemm, Christian H. Nolte, Matthias Endres

[**1. Supplementary Methods** 2](#_Toc4267365)

[1.1 Real-world geographic stroke care infrastructure environments 2](#_Toc4267366)

[1.2 Mathematical definition of triage regions 3](#_Toc4267367)

[1.3 Summary of technical steps of the simulation 4](#_Toc4267368)

# **1. Supplementary Methods**

## 1.1 Real-world geographic stroke care infrastructure environments

To investigate the importance of considering additional primary stroke centers as transport destinations during prehospital triage decisions for acute ischaemic stroke patients, we performed analyses in two geographic scenarios that were based on real-world stroke care infrastructures: For a metropolitan environment, the city of Berlin, Germany; for a rural/mixed environment, the state of Brandenburg.

The table contains key geographic and demographic parameters for both regions. Figure S1 visualizes locations of the stroke centers. For the Berlin region, endovascular therapy is offered at most certified stroke units. For our analysis to be more reflective of a typical urban infrastructure, mechanical thrombectomy was assumed to be available at the three campuses of the University hospital. For Berlin and Brandenburg, intravenous thrombolysis was assumed to be available at all certified stroke units and, in addition, at hospitals that are administering intravenous thrombolysis to stroke patients after tele-neurological consultation. The choice of availability of intravenous thrombolysis and mechanical thrombectomy did not have the goal to be as close to reality as possible, but to develop a model with characteristics of urban and rural geographic environments, in which findings that were obtained in abstract geographic environments could be validated.

| **Supplementary Table 1.** Characteristics of real-world stroke care infrastructure environments | | |
| --- | --- | --- |
| **Region** | **Berlin** | **Brandenburg** |
| Area (km²) | 892 | 29,654 |
| Population | 3,601,131 | 2,494,648 |
| Population density (inhabitants per km²) | 4,039 | 84 |
| Number of CSCs in the model | 3 | 7 |
| Number of PSCs in the model | 11 | 24 |


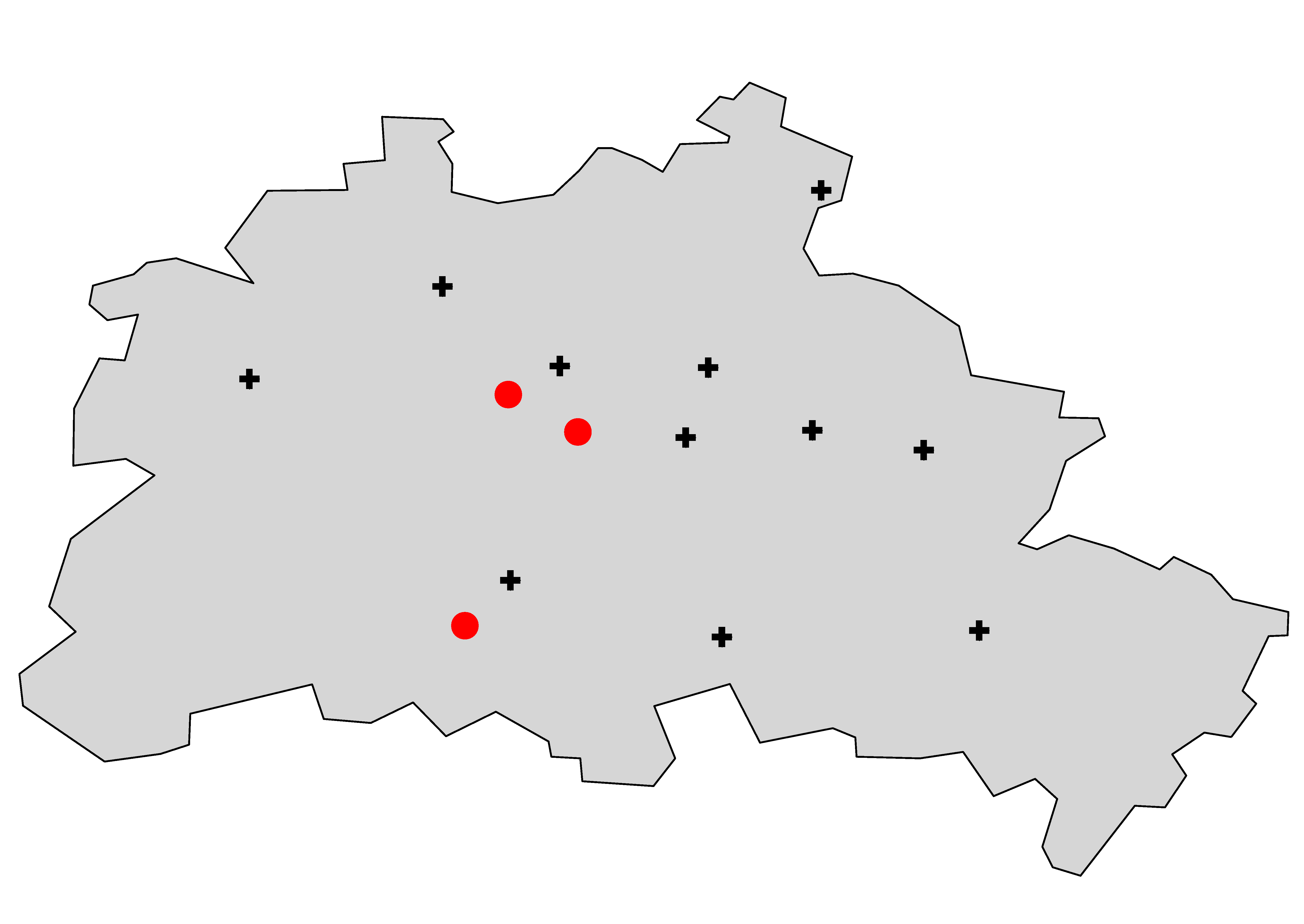

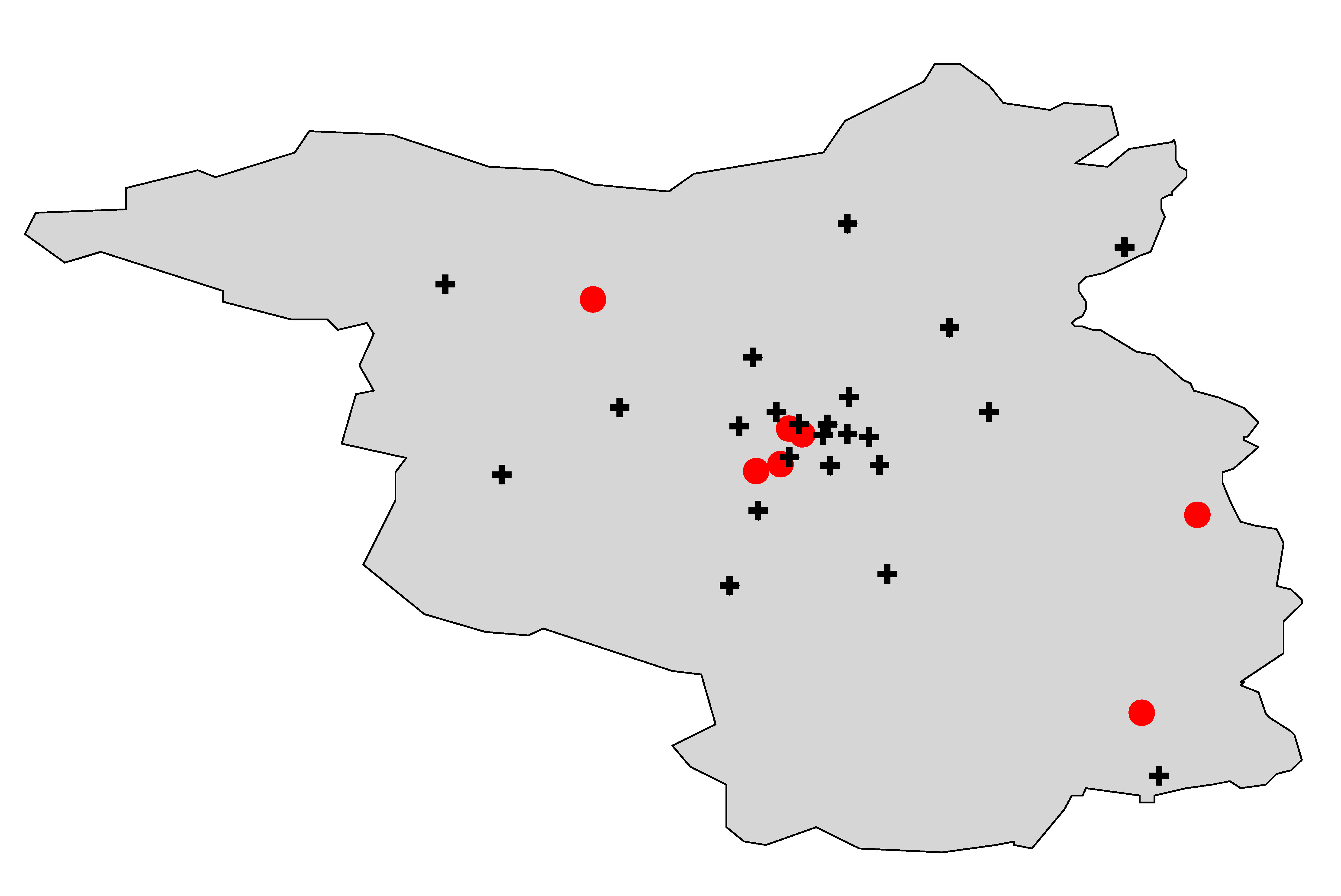


**Supplementary Figure 1.** Location of comprehensive and primary stroke centers in the urban and rural model based on the stroke care infrastructure environments of Berlin and Brandenburg.

Left: Berlin, right: Brandenburg. Black crosses represent primary stroke centers, red circles comprehensive stroke centers.

## 1.2 Mathematical definition of triage regions

For the random abstract as well as for the real-world geographic scenarios, sub-regions within the incident region were defined according to specific patterns of travel times from the points within the sub-region to the CSCs and PSCs. For any given point within the incident region *U*, let dCSC_i_ and dPSC_j_ denote the time to the *i*th CSC and *j*th PSC, respectively. Additionally, let dTransfer_j_ denote the time from the *j*th PSC to its nearest CSC. The unconditional catchment region of the CSC, where only one reasonable transport destination exists, is then defined as:

$A_{1}=\left\{ \left( x,y \right)\in U |\min_{i\in[1,2,\ldots,4]} {dCSC}_{i}\leq\min_{j\in[1,2,\ldots,10]} {dPSC}_{j} \right\}$.

The remaining points constitute the region where more than one transport options exist and a triage decision is required:

$A_{>1}=U\setminus A_{1}$.

This triage region $A_{>1}$ is further sub-divided according to the number of transport destinations that need to be considered for an exhaustive triage decision. Potential transport destinations include the nearest CSC, the nearest PSC, and, in addition, any of the remaining PSCs that are not closest to the scene, but transport to which would imply a shorter total time-to-CSC-via-PSC as compared to transport to any other PSC that is closer to the scene (**Figure 1,** main text). In mathematical terms, the number of PSCs η that need to be considered as potential transport destinations at any given point $\left( x,y \right)$ within the triage region $A_{>1}$ can be expressed as:

$$\eta_{\left( x,y \right)}=\#\left\{ k | (\nexists j )[\left( \mathrm{dPSC}_{j}<\mathrm{dPSC}_{k} \right) \wedge\left( {\mathrm{dPSC}_{j}+dTransfer}_{j}<{\mathrm{dPSC}_{k}+dTransfer}_{k} \right)] \right\};$$

$k,j\in\left[ 1, \ldots, 10 \right]$,

where # denotes the cardinality of the set. Accordingly, the sub-divisions of the triage region $A_{>1}$ are defined as

$A_{m}=\left\{ \left( x,y \right)\in A_{>1} | \eta_{\left( x,y \right)}=m-1 \right\}; m=2, 3, \ldots$,

and the *higher order triage region* where more than two triage options need to be considered as

$$A_{>2}= \bigcup_{m>2} A_{m}.$$

## 1.3 Summary of technical steps of the simulation

In the following, we provide a list-wise summary of the technical steps involved in the project. Details are explained in the main manuscript and in previous sections of this Online Supplement

Steps were similar for abstract geographic environments and real-world geographic scenarios. For the abstract scenarios, the simulation involved random manifestations of scenarios with different numbers of PSCs and CSCs (Loop 1 and Loop 2).

Abstract geographic environments:

1. Choose combination of total number of PSCs (2 – 10) and CSCs (1 – 4)
2. Randomly position PSCs and CSCs in the disc (homogenous spatial Poisson point process)
3. Calculate driving times between points in the disc and between stroke centers as the Euclidean distance between points
4. Determine relative size of sub-regions (primary catchment area of CSCs, triage region, higher order triage region) as outlined in [1.2 Mathematical definition of triage regions]
5. Calculate benefit/harm ratios of each location as explained in the main text, section 2.1, and in Figure 1 in the main text. Calculate spatial median of benefit/harm ratios
6. Loop 1: Repeat previous steps 2 – 5 a total of n = 50 times and use results to calculate distributions of the relative sizes of the triage sub-regions (Figure 3 in the main text) and distributions of the spatial median of the benefit/harm ratio (Figure 4 in the main text)
7. Loop 2: Choose different combination of total number of PSCs and CSCs (see step 1) and repeat steps 1 – 6

Real-world geographic scenarios

1. Determine positions of PSCs and CSCs based on real-world infrastructure in Berlin and Brandenburg (see Supplementary Figure 1)
2. Calculate driving times between points in the area and between stroke centers as the haversine distance between points, multiplied by a proportionality factor derived from the Google Maps Distance Matrix API
3. Determine sub-regions and spatial distributions of benefit/harm regions (Figure 5 in the main text)
